# Supplementary material for: Ferrocene-Based Terpolyamides and Their PDMS-Containing Block Copolymers: Synthesis and Physical Properties
Source: Polymers (Basel). 2022 Nov 23;14(23):5087. doi: 10.3390/polym14235087 (PMC9735706; doi:10.3390/polym14235087)
Supplement: Supplementary file 1 [file polymers-14-05087-s001.zip › polymers-2005131-supplementary.pdf]

Supplementary information

Ferrocene-Based Terpolyamides and their PDMS Containing Block Copolymers: Synthesis and Physical Properties

Irrum Mushtaq,[1,3*] Erum Jabeen,[2] Zareen Akhter,[3*] Fatima Javed,[4] Azfar Hassan,[5] Muhammad Saif Ullah Khan,[3] Faheem Ullah,[1,6] Faiz Ullah Shah[7*]

[1]Department of Biological Sciences, National University of Medical Sciences, The Mall road, Rawalpindi, 46000, Pakistan

[2]Department of Chemistry, Faculty of Science, Allama Iqbal Open University, Islamabad, 44310, Pakistan

[3]Department of Chemistry, Quaid-i-Azam University, Islamabad, 45320, Pakistan

[4]Department of Chemistry, Shaheed Benazir Bhutto Women University, Peshawar, 25000, Pakistan

[5]Department of Chemical and Petroleum Engineering, University of Calgary, Calgary, AB, T2N 1N4 Canada

[6]School of Materials and Mineral Resources Engineering, Engineering Campus, University Sains Malaysia Nibong Tebal, 14300, Palau Penang, Malaysia

[7]Chemistry of Interfaces, Luleå University of Technology, SE-971 87 Luleå, Sweden

Corresponding authors:

Irrum Mushtaq
Irrum.mushtaq@numspak.edu.pk

Zareen Akhter
Zareen_a@qau.edu.pk

Faiz Ullah Shah
faiz.ullah@ltu.se

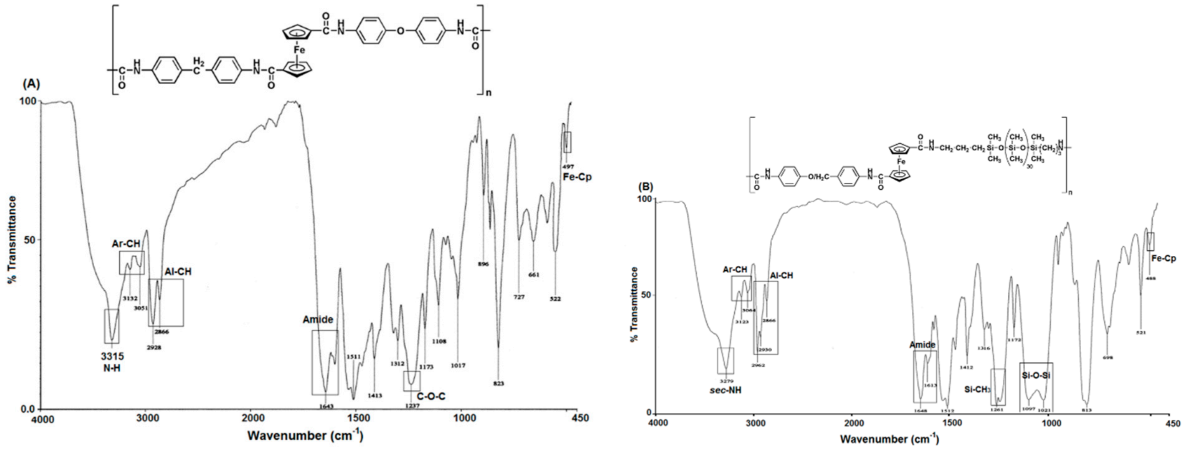

Figure S1. Representative FTIR spectra. (A) Terpolyamide (F2), and (B) block copolymer (PF2).

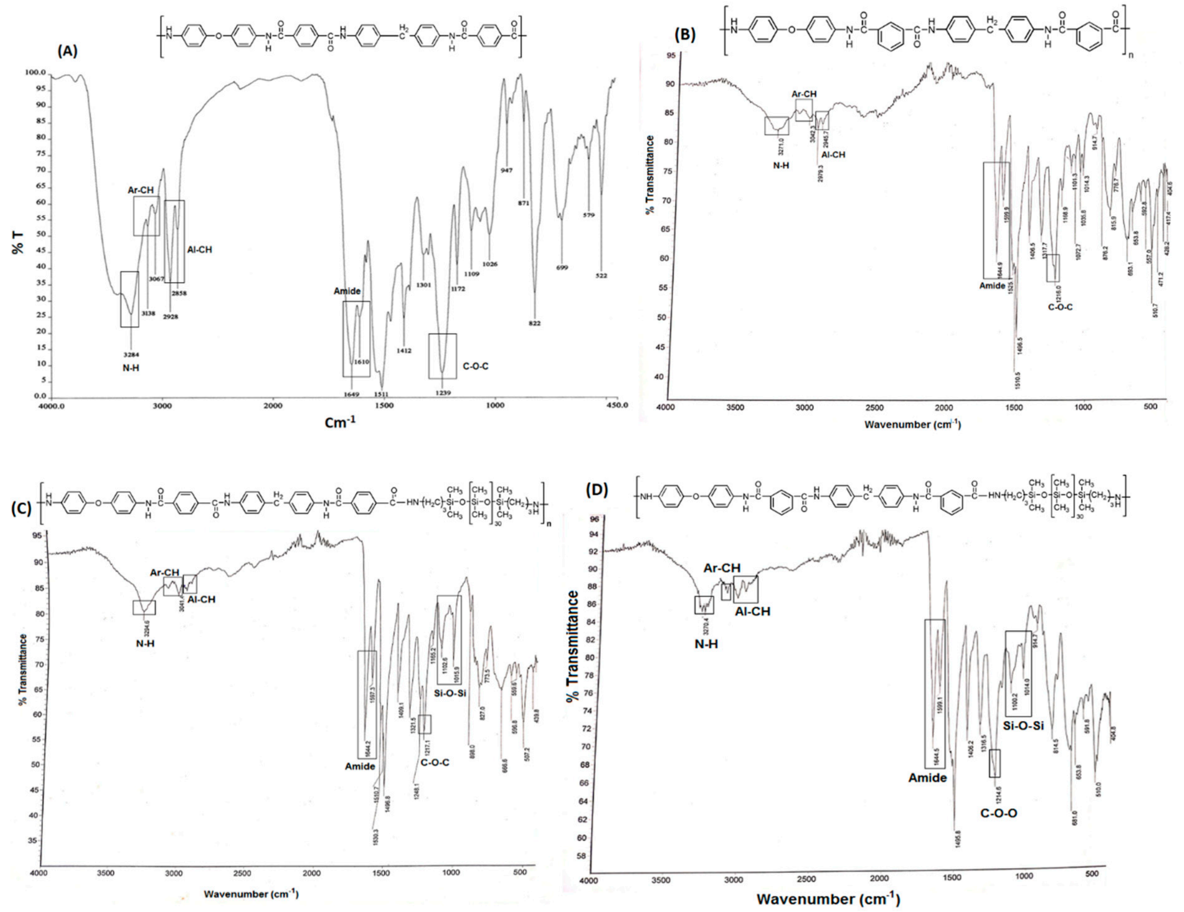

Figure S2. FTIR spectra of representative terpolymers (A) T2, and (B) I2 and their PDMS containing block copolymers (C) PT2, and (D) PI2.

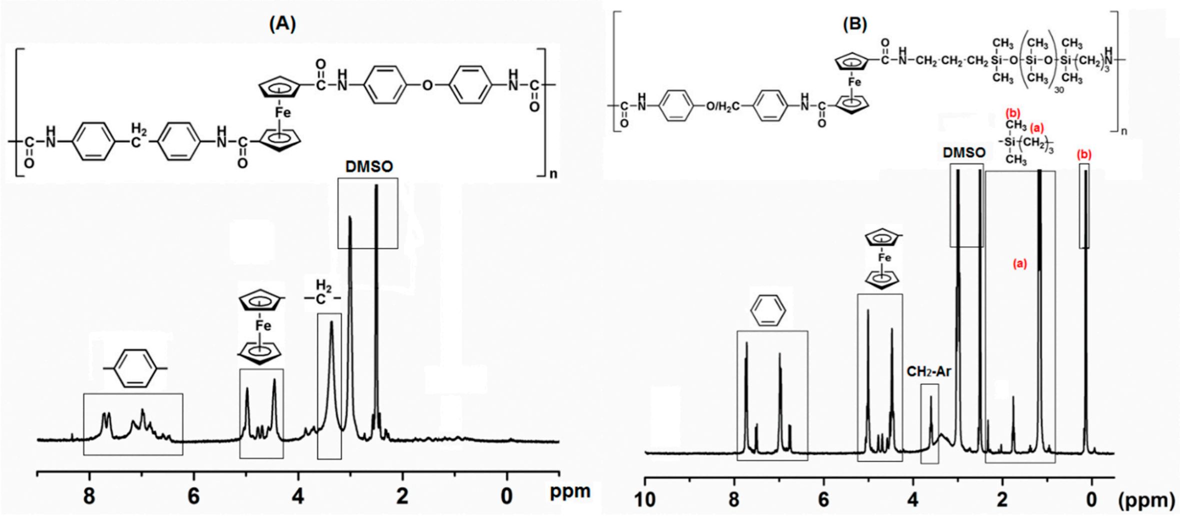

Figure S3. ^{1}H NMR spectra. (A) Ferrocene-based terpolyamide (F2), (B) Ferrocene-based PDMS containing block copolymer.

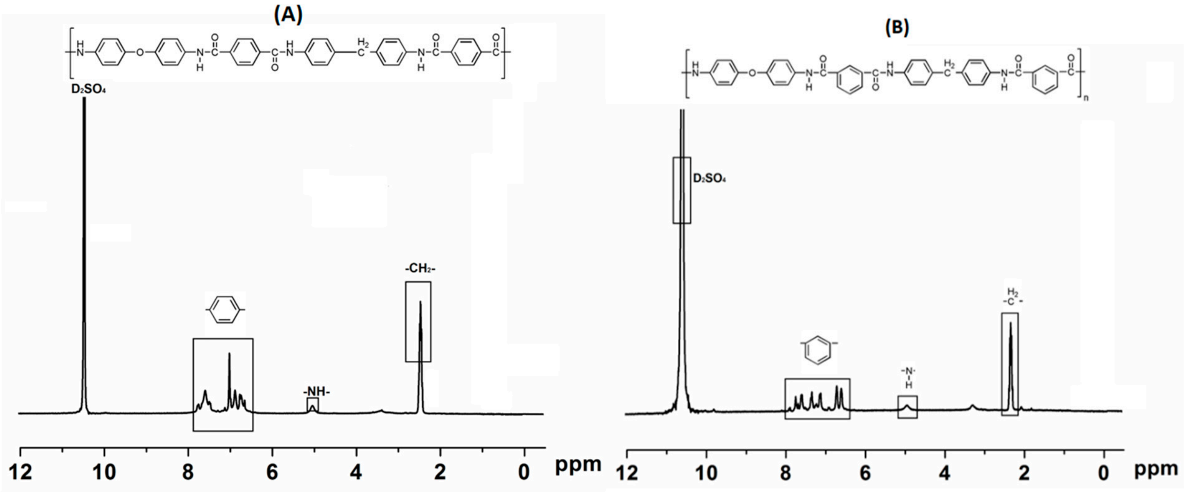

Figure S4. ^{1}H NMR spectra of the representative organic analogues (A) T2 and (B) I2.

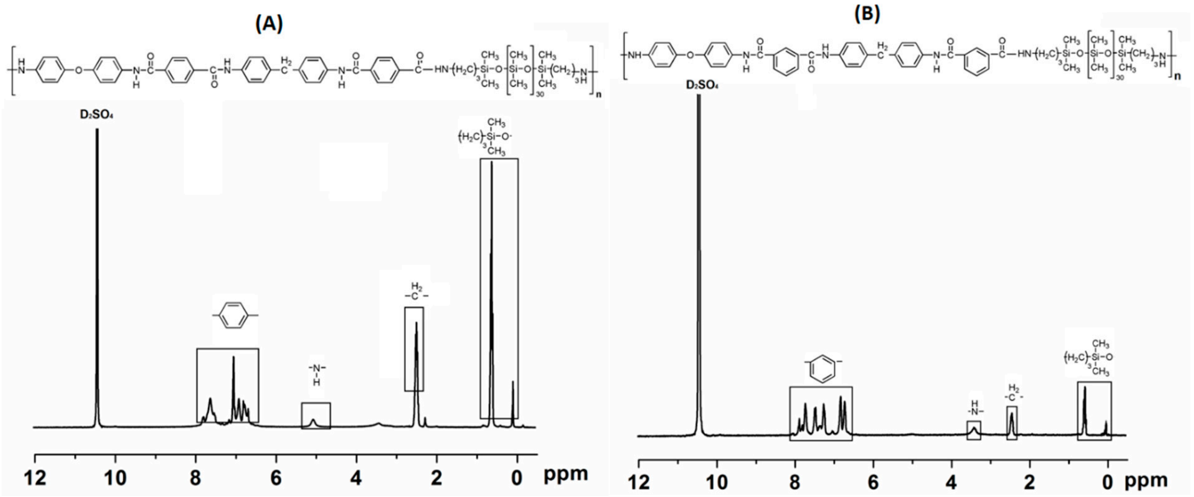

Figure S5. ^{1}H NMR spectra of the representative organic analogues of PDMS containing block copolymers (A) PT2 and (B) PI2.

Table S1. Computed HOMO and LUMO spread of small monomeric units computed at DFT/B3LYP/6-311G**(d, p) basis set.

Codes	Structures	HOMO	LUMO
M1	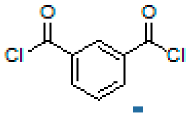	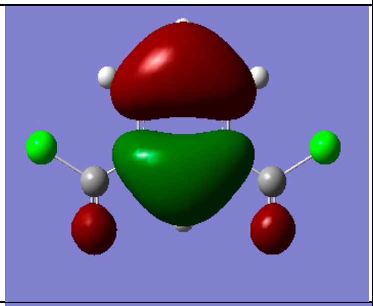	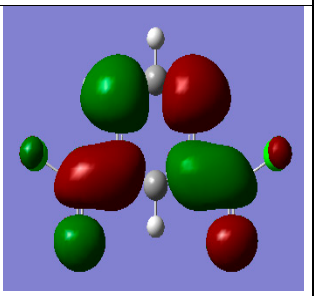
M1'	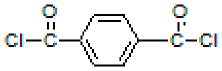	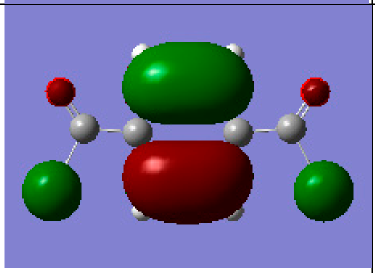	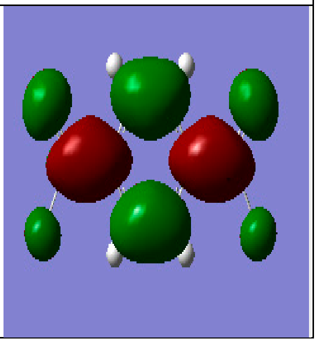

M2	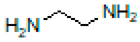	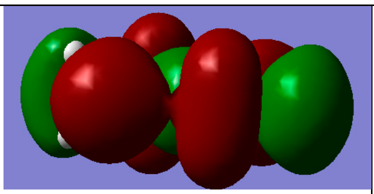	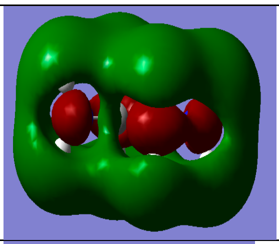
M3	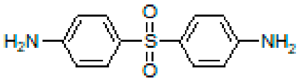	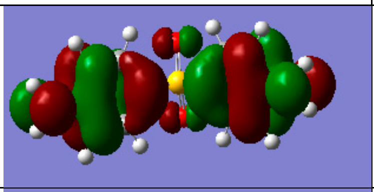	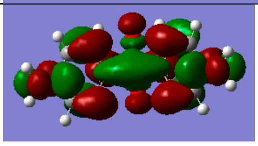
M4	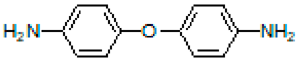	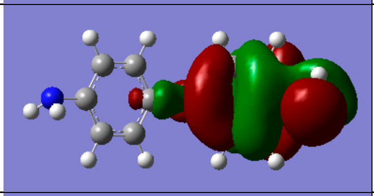	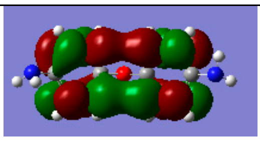
M5	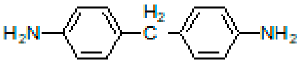	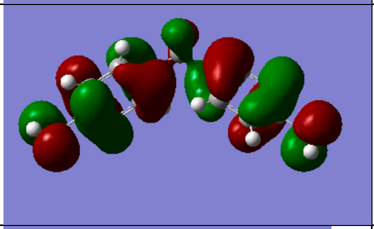	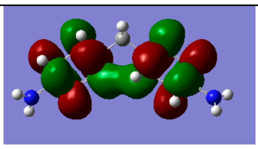
M6	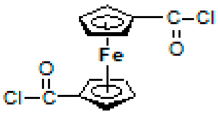	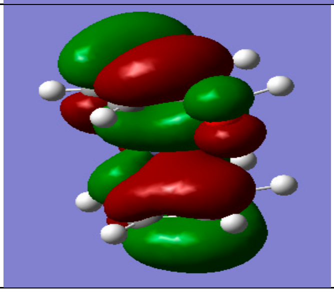	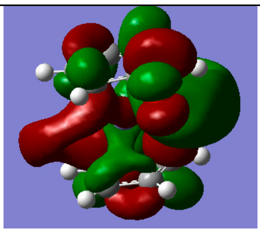
